# Safety and efficacy of immune checkpoint inhibitors (ICIs) in cancer patients with HIV, hepatitis B, or hepatitis C viral infection

**DOI:** 10.1186/s40425-019-0771-1

**Published:** 2019-12-17

**Authors:** Neil J. Shah, Ghassan Al-Shbool, Matthew Blackburn, Michael Cook, Anas Belouali, Stephen V. Liu, Subha Madhavan, Aiwu Ruth He, Michael B. Atkins, Geoffrey T. Gibney, Chul Kim

**Affiliations:** 1 Lombardi Comprehensive Cancer Center MedStar Georgetown University Hospital, 3800 Reservoir Rd. N.W., LCCC Bldg, 2nd FL, Pod B P413, Washington, DC 20007 USA; 2 Memorial Sloan Kettering Cancer Center, Manhattan, New York City USA; 3 Department of Medicine, MedStar Washington Hospital Center, Washington, DC USA; 4 Department of Medicine, MedStar Georgetown University Hospital, Washington, DC USA; 5 Innovation Center for Biomedical Informatics (ICBI), Georgetown University, Washington, DC USA

**Keywords:** Immune checkpoint inhibitors (ICI), Human immunodeficiency virus (HIV), Hepatitis B (HBV), Hepatitis C (HCV), Immune related adverse events (irAEs)

## Abstract

**Background:**

Patients with chronic viral infections including human immunodeficiency virus (HIV), hepatitis B (HBV) and hepatitis C (HCV) are at increased risk of developing malignancies. The safety and efficacy of ICI therapy in patients with both cancer and chronic viral infections is not well established as most clinical trials of ICIs excluded these patient populations.

**Methods:**

We performed a retrospective analysis of patients with advanced-stage cancers and HIV, HBV, or HCV infection treated with ICI therapy at 5 MedStar Health hospitals from January 2011 to April 2018.

**Results:**

We identified 50 patients including 16 HIV, 29 HBV/HCV, and 5 with concurrent HIV and either HBV or HCV. In the HIV cohort (*n* = 21), any grade immune-related adverse events (irAEs) were 24% with grade ≥ 3 irAEs 14%. Among 5 patients with matched pre/post-treatment results, no significant changes in HIV viral load and CD4+ T-cell counts were observed. RECIST confirmed (*n* = 18) overall response rate (ORR) was 28% with 2 complete responses (CR) and 3 partial responses (PR). Responders included 2 patients with low baseline CD4+ T-cell counts (40 and 77 cells/ul, respectively). In the HBV/HCV cohort (*n* = 34), any grade irAEs were 44% with grade ≥ 3 irAEs 29%. RECIST confirmed ORR was 21% (6 PR). Among the 6 patients with known pre/post-treatment viral titers (2 HCV and 4 HBV), there was no evidence of viral reactivation.

**Conclusions:**

Our retrospective series is one of the largest case series to report clinical outcomes among HIV, HBV and HCV patients treated with ICI therapy. Toxicity and efficacy rates were similar to those observed in patients without chronic viral infections. Viral reactivation was not observed. Tumor responses occurred in HIV patients with low CD4 T-cell counts. While prospective studies are needed to validate above findings, these data support not excluding such patients from ICI–based clinical trials or treatment.

## Background

Cancer immunotherapy is transforming the way we treat patients with cancer. Immune checkpoint inhibitor (ICI) therapy is a type of cancer immunotherapy that works through suppression of immune inhibitory pathways such as the programmed cell death protein-1 (PD-1)/programmed death-ligand-1 (PD-L1) axis and the cytotoxic lymphocytes antigen proteins (CTLA-4) pathway [1]. The impressive outcomes with ICI therapy in clinical trials led to approval of several ICIs by the U.S. Food and Drug Administration (FDA) in multiple advanced malignancies. For example, for the first-line treatment of patients with non-small-cell lung cancer (NSCLC) without actionable alterations, ICI therapy, either alone or in combination with chemotherapy improves survival compared to chemotherapy alone and is now considered standard of care [2–5]. Similarly, ICI therapy has improved outcomes in patients with melanoma [6, 7], renal cell carcinoma (RCC) [8–10] and many other cancers [11]. The majority of early clinical trials of ICIs excluded patients with chronic viral infections such as human immune-deficiency virus (HIV), hepatitis B virus (HBV), and hepatitis C virus (HCV) due to concerns about viral reactivation, toxicity, and efficacy in these populations.

Limited data from the literature exist on the safety and efficacy of ICI therapy in patients with chronic viral infection and advanced-stage cancer. A clinical trial of the anti-PD-1 antibody pembrolizumab in patients with HIV on anti-retroviral therapy and advanced-stage cancer, reported that pembrolizumab did not impair CD4+ cell counts or viral suppression [12, 13]. Likewise, a systematic review showed that ICI therapy was not associated with new safety signals in patients with HIV infection and advanced-stage cancer [14]. Although a few case studies reported HBV reactivation upon ICI therapy [15, 16], clinical trials of ICI therapy in patients with hepatocellular carcinoma (HCC) did not show evidence of reactivation of HBV/HCV [17, 18]. While reassuring, these analyses involve small patient numbers and the treatment was mainly limited to ICI monotherapy. In order to shed further light on the safety and efficacy of ICI therapy in patients with concomitant cancer and chronic viral infections, we performed a retrospective analysis of cancer patients with chronic viral infection (HIV, HBV, or HCV) who were treated with ICI containing regimens including chemotherapy plus ICI therapy.

## Methods

We have developed a comprehensive REDCap based immuno-oncology database (IO database) at MedStar Health Hospitals to capture real world data of patients treated with ICI therapy. Pharmacy records were used to identify patients treated with either anti-PD (L)-1 (nivolumab, pembrolizumab, atezolizumab, durvalumab and avelumab), anti-CTLA-4 (ipilimumab) as a single agent or in combination with other ICIs (ipilimumab plus nivolumab) or chemotherapy/targeted therapy [carboplatin plus pemetrexed plus pembrolizumab (carbo/pem/pembro), carboplatin plus paclitaxel plus pembrolizumab (carbo/taxol/pembro) and brentuximab plus nivolumab]. In this database, we have collected a total of 769 patients treated at 5 MedStar Health hospitals (MedStar Georgetown University Hospital, MedStar Washington Hospital Center, MedStar Franklin Square Hospital, MedStar Good Samaritan Hospital, and MedStar Union Memorial Hospital) during the time frame of January 2011 to April 2018. A total of 50 patients with chronic viral infections (HIV, HBV and/or HCV) were identified from the database. Patient’s HIV/HBV/HCV status was attained based on ICD-9/10 codes and manual review of medical chats which was performed for each patient. HCV patients in virologic remission after therapy were included. Objective response rate (ORR) was measured using RECIST version 1.1 criteria [19]. The patients without any follow-up scans either due to clinical deterioration or lost to follow up were assumed to progressive disease (PD) as best ORR. Two patients with Hodgkin’s lymphoma (HD) and 1 patient with Burkitt’s lymphoma were not included in response evaluation since RECIST is not the standard response criteria used for lymphoma. CTCAE version 4.03 was used to grade immune-related adverse events (irAEs). Bio-informatics support was used to abstract patients’ demographics, co-morbidities, treatment history, and toxicity from electronic medical records. Data was extracted using SQL queries. R and Python programming was used for data cleansing, calculations, code mapping, and aggregation. Patients’ RECIST confirmed response and toxicity were verified for each patient by the investigators. Additional data collected manually included HIV viral load, CD4+ T-cell counts, HIV medication history, HCV viral load, HCV treatment history, HBsAg, HBsAb, HBcAb, HBeAb, HBV viral load and HBV treatment if available. Pre-treatment values were defined as any values obtained before the first dose of ICI therapy and post-treatment values as any values obtained after the first dose of ICI therapy. A low CD4+ T-cell count was defined as < 100 cells/ul. Descriptive statistics were applied to summarize the data. Tumors samples were classified as PD-L1+ if PD-L1 expression was noted in ≥1% of tumor cells using the Dako PD-L1 IHC 22C3 PharmDx clone (*n* = 9) or the VENTANA PD-L1 (SP-142) (*n* = 1) assay.

## Results

We identified 50 patients with HIV, HBV and HCV co-morbidities. Table 1 outlines patient infections and co-infections. Clinical characteristics and tumor types are presented in Table 2. The median age of patients in both the HIV and HBV/HCV cohorts was 62 years. The majority of patients were treated with anti-PD-(L)1 monotherapy (*n* = 43). One patient received combination ipilimumab and nivolumab and 6 patients were treated with anti-PD-(L)1/chemotherapy/targeted therapy combination (4 with carbo/pem/pembro). The most common type of cancer in the HIV cohort was NSCLC (57%, *n* = 12). HCC (47%, *n* = 16) was the most common type of cancer in the HBV/HCV cohort, followed by NSCLC (29%, *n* = 10).

**Table 1:** Classification of HIV, HBV, and HCV infections

Infections	Number of patients (*N* = 50)
HIV	16
HBV	10
HCV	15
HIV/HBV co-infection	1
HIV/HCV co-infection	3
HBV/HCV co-infection	4
HIV/HBV/HCV co-infection	1

**Table 2:** Patients’ characteristics, tumor type and ICI treatments

Baseline Characteristics	HIV (*N* = 21)	HBV/HCV (*N* = 34)
Age – Median (range)	62 (29–85)	62 (29–79)
Sex (% Male)	52%	71%
Race		
(White %)	33	25
(African American %)	67	50
(Asian%)	0	12
Tumor types		
Non-small cell lung cancer (NSCLC)	12	10
Adenocarcinoma	8	5
Squamous	2	1
Non specified	2	4
Anal squamous cell carcinoma	2	2
Hodgkin’s lymphoma (HD)	2	0
Head and neck squamous cell carcinoma (H&N)	1	1
Colorectal carcinoma (CRC)	1	0
Burkitt lymphoma	1	0
Renal clear cell carcinoma (RCC)	1	3
Hepatocellular carcinoma (HCC)	1	16
Small cell lung cancer (SCLC)	0	1
Gastric cancer	0	1
ICI therapy type		
Anti-PD-(L)1 monotherapy	16	30
Anti-PD-(L)1 in combination with anti-CTLA-4	0	1
Anti-PD-(L)1 in combination with chemotherapy	5	3

### Safety and efficacy of ICI therapy in patients with HIV

Among 21 HIV patients, baseline CD4+ T-cell counts were available in 16 patients before initiation of ICIs (4 with < 100 cells/ul, 4 with < 200 cells/ul and 8 with ≥200 cells/ul); 5 patients had CD4+ T-cell counts within 1 month before starting ICIs. CD4+ T-cell counts were available in 12 patients at any point during or after stopping ICIs therapy. Among 5 patients with both pre-treatment and post-treatment CD4+ T-cell counts, 2 treated with PD-1 monotherapy and 3 with ICI plus chemotherapy, no significant changes were noted (Additional file 1: Table S1). Two patients with low CD4+ T-cell counts remained low and 3 with high CD4+ T-cell counts remained high. Pre-treatment HIV viral load was available in 15 patients with 6 patients having HIV viral load within 1 month of ICI initiation. Among these 6 patients, 4 had an undetectable viral load and 2 had high viral loads of 111,000 copies/ml and 56,572 copies/ml, respectively at ICI initiation. Of these 6 patients, only 5 patients had both a pre- and post-treatment HIV viral loads of which two patients maintained undetectable levels, one patient’s viral load increased from 0 to 81 copies/ml, and two patients’ viral load decreased (111,000 to 7960 copies/ml and 56,572 to 82 copies/ml). HIV treatment history was available in 13 patients [Tenofovir and emtricitabine (truvada) plus raltegravir (isentress) (2), tenofovir alafenamide and emtricitabine (descovy) plus raltegravir (isentress) (1), tenofovir alafenamide and emtricitabine (descovy) plus dolutegravir (tivicay) (2), tenofovir alafenamide and emtricitabine (descovy) plus darunavir (prezista) (1), tenofovir alafenamide and emtricitabine (descovy) plus darunavir (prezista) plus ritonavir (norvir) (1), elvitegravir plus cobicistat plus emtricitabine plus tenofovir alafenamide (genovoya) (2), bictegravir plus emtricitabine plus tenofovir alafenamide (biktarvy) (1), emtricitabine plus rilpivirine plus tenofovir disoproxil fumarate (complera) (2), and raltegravir (isentress) plus nevirapine (viramune) plus lamivudine (epivir) (1)]. In the two patients with decrease in HIV load after ICI therapy, it was noted that they became more compliant to their HIV treatment.

The incidence of irAEs among the HIV cohort (*n* = 21) of any grade was 24% (*n* = 5) and grade ≥ 3 was 14% [*n* = 3; hepatitis (*n* = 1) and pneumonitis (n = 2)] (Table 3). All grade ≥ 3 or higher irAEs were noted in patients treated with anti-PD-1 monotherapy and both patients who developed grade 3 pneumonitis were being treated for NSCLC. Among the 5 HIV patients who developed any grade irAEs, 1 had low CD4+ T-cell counts during ICI treatment. The risk of irAEs did not seem to increase with the addition of chemotherapy to anti-PD-(L)1 therapy.

**Table 3:** Safety and efficacy analysis of HIV and HBV/HCV cohorts

Cohorts (N)	ORR^a^ N (%)	Any Grade irAEs N (%)	Grade ≥ 3 irAEs N (%)
HIV (21)	2 CR/3 PR (28)	5 (24)	3 (14)
HBV/HCV (34)	6 PR (18)	15 (44)	10 (29)^b^

^a^Response evaluable patients HIV: 18; HBV/HCV: 34, ^b^ Two patients with baseline grade ≥ 2 hepatitis

**Fig. 1:**
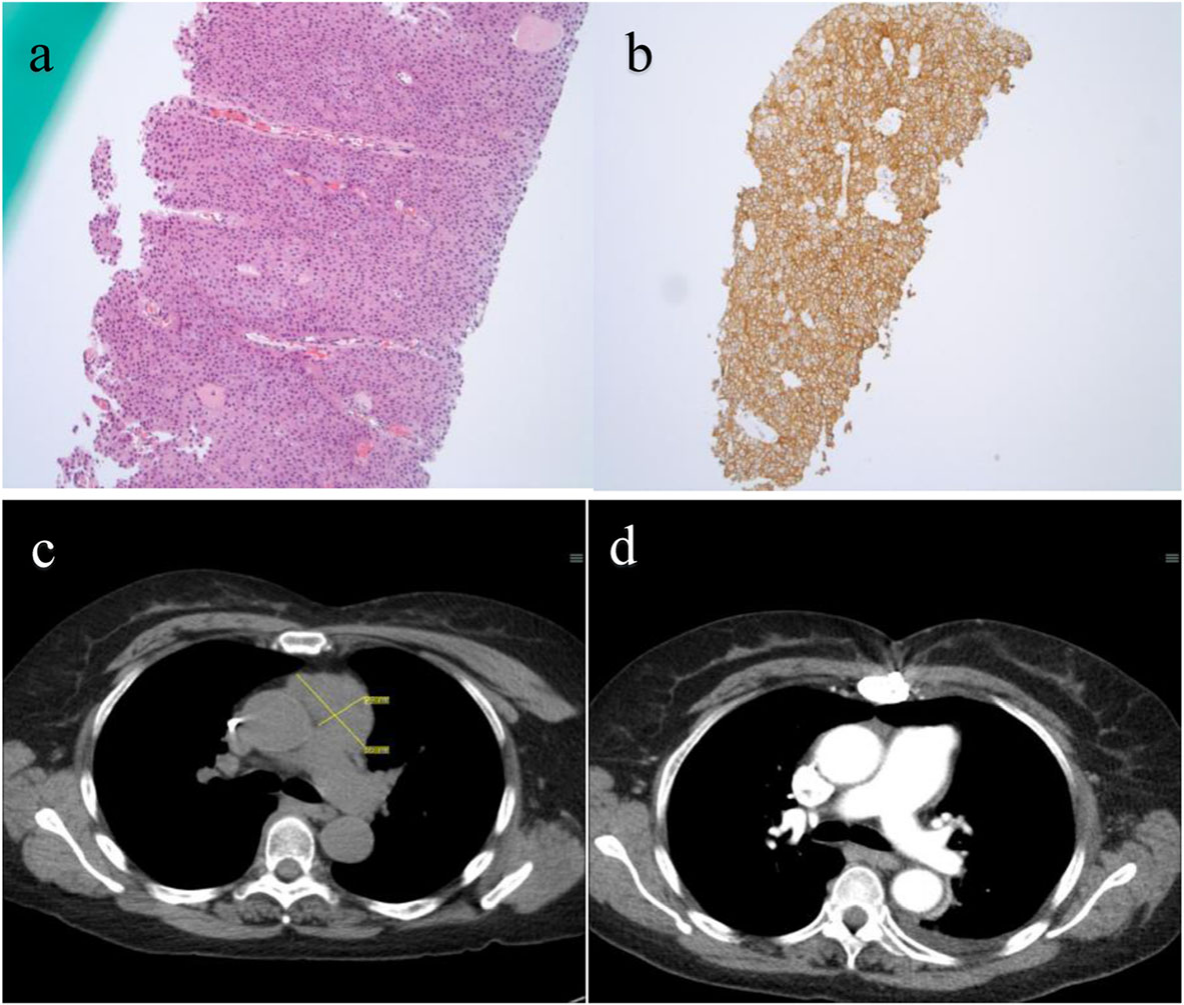
Pathology and Radiology findings or patient with low CD-4+ T-cell count (40 cells/ul). **a**. core biopsy of mediastinal mass suggestive of invasive squamous cell carcinoma. **b**. PD-L1 by IHC - 100% +. **c**. Pre-treatment CT chest suggestive of 5.5 × 2. 9 cm mediastinal mass. d. CT chest suggestive of CR with ICI therapy

Among RECIST evaluable patients (*n* = 18), the ORR was 28% with two complete responses (CR) and 3 partial responses (PR). Among responders, pre-treatment CD4+ T-cell count were available in 3 patients, two had low counts (40 cells/ul and 77 cells/ul) and one patient with CD4+ T-cell count of 616 cells/ul. A complete response was seen in patients with NSCLC and microsatellite instability high colorectal cancer (CRC) treated with anti-PD-1 monotherapy. The ORR was 13% among 8 NSCLC patients treated with anti-PD-1 monotherapy and 75% among 4 NSCLC patients treated with anti-PD-1 and chemotherapy combination. Tumor PD-L1 status was available in 9 patients, of which 7 were PD-L1 positive. The ORR in PD-L1 positive patients treated with anit-PD-1 monotherapy (*n* = 5) was 20% (1 CR) and 100% with anti-PD-1 plus chemotherapy (*n* = 2). One patient who had a CR with anti-PD-1 monotherapy had PD-L1 expression of 100% and a pretreatment CD4+ T-cell count was 10 and 40 cells/ul (1 year and 1 month before starting ICI treatment) with post treatment CD4+ T-cell count of 67cell/ul. Pathology and radiology findings of this patient are shown in Fig. 1.

### Safety and efficacy of ICI therapy in patients with HBV/HCV

Among 23 HCV patients (18 HCV and 5 HBV/HCV), 9 patients were successfully treated for their HCV infection, 9 patients were untreated and 5 patients had unknown treatment status before ICI therapy initiation. Among the 9 untreated HCV patients, none received HCV treatment concurrently with ICI treatment. Among 16 HBV patients (11 HBV and 5 HBV/HCV), 8 patients had positive HBsAg, 4 patients were HBsAg (−), HBsAb (−), and HBcAb (+), and 3 patients were HBsAg (−), HBsAb (+), and HBcAb (+). One patient’s HBV status was unknown. Pre-treatment HBV viral loads were available in 13 patients with 8 patients having undetectable HBV viral titer and the remaining 5 with detectable viral loads (39 IU/ml, 10 IU/ml, 250 IU/ml, 92 IU/ml, and 77 IU/ml). Pre- and post-treatment viral loads were available in 4 patients and HBV viral load remained undetectable in all these patients. Nine patients were taking anti-HBV treatment [Tenofovir (6)/entecavir (3)] during ICI treatment and no changes in anti-HBV medication were made during ICI treatment.

In the combined HCV/HBV group, any grade irAEs were noted in 44% (*n* = 15) and grade ≥ 3 in 29% (*n* = 10) (Table 3). The individual irAEs were colitis 12% (*n* = 4), skin rash/pruritus 18% (*n* = 6), hepatitis 18% (n = 6), pneumonitis 6% (*n* = 2), hypothyroidism 6% (n = 2) and one patient with diabetes mellitus and encephalitis. Grade ≥ 3 irAEs were colitis (*n* = 3), hepatitis (n = 4), diabetes (n = 1), rash (n = 1) and pneumonitis (n = 1); one patient had baseline grade 2 hepatic enzyme elevation which progressed to grade 3 and one patient had baseline grade 3 hepatic enzyme elevation which progressed but remained grade 3. No HBV viral reactivation or changes in HBV medications were observed in any patients.

Among RECIST evaluable patients (*n* = 34), the ORR for the combined HCV/HBV cohort was 18% (6 PR) (Table 3). The ORR for HCV patients (*n* = 23) was 17% (4PR, 5 SD and 14 PD). Among HCV patients who demonstrated response to ICIs, 3 patients were previously treated for HCV. The ORR for HBV cohort (*n* = 16) was 25% (4 PR, 3 SD and 9 PD).

### Safety according to type of ICI therapy in patient with HIV, HBV and HCV

We identified 16 patients with HIV and 30 patients with HBV/HCV who were treated with anti-PD-(L)1 monotherapy and 5 HIV and 3 HBV/HCV patients treated with chemotherapy plus ICI (Table 4). One SCLC patient with HBC/HCV received treatment with anti-PD-1 and anti-CTLA-4 combination ICI therapy and developed grade 2 colitis and grade 3 pneumonitis. Among HIV patients treated with anti-PD-(L)1 monotherapy, the incidence of any grade irAEs was 25% (hepatitis, rash, pneumonitis and hypothyroidism, *n* = 2 each) and grade ≥ 3 irAEs were 19% [pneumonitis (*n* = 2) and hepatitis (*n* = 1)]. The incidence of any grade irAEs in the HBV/HCV cohort treated with anti-PD-(L)1 monotherapy was 43% with skin rash/pruritus (*n* = 6), and hepatitis (*n* = 6) being the most common and grade ≥ 3 irAEs were 27% [colitis (n = 2), hepatitis (*n* = 4), diabetes mellitus and rash, n = 1 each (two patients with baseline ≥2 hepatitis)].The incidence of any grade irAE in the HIV and HBV/HCV cohorts treated with ICI-chemotherapy combinations was 20 and 33%, respectively with one patient who developed grade 3 colitis in the HBV/HCV cohort.

**Table 4:** Subgroup analysis of safety according to ICI therapy types

	Anti-PD (L)-1 Mono-Therapy				Anti-PD (L)-1 plus Chemotherapy			
	HIV (16)		HBV/HCV (30)		HIV (5)		HBVC/HCV(3)	
	Any Grade irAEs N (%)	Grade ≥ 3 irAEs N (%)	Any Grade irAEs N (%)	Grade ≥3 irAEs N (%)	Any Grade irAEs N (%)	Grade ≥ 3 irAEs N (%)	Any Grade irAEs N (%)	Grade ≥ 3 irAEs N (%)
Total	4 (25)	3(19)	13(43)^a^	8(27)^a^	1(20)	0	1(33)	1(33)
Colitis	0	0	2	2	0	0	1	1
Hepatitis	2	1	6	4	0	0	0	0
Rash	2	0	6	1	1	0	0	0
Hypothyroidism	2	0	1	0	1	0	0	0
Pneumonitis	2	2	1	0	0	0	0	0
Arthritis	0	0	2	0	0	0	0	0
Diabetes Mellitus	0	0	1	1	0	0	0	0
Encephalitis	0	0	1	0	0	0	0	0

^a^Two patients with baseline grade ≥ 2 hepatitis

### Safety and efficacy of ICI therapy according to the tumor type in patients with HIV, HBV and HCV

The predominant tumor type in the HIV cohort was NSCLC (*n* = 12) including 8 patients treated anti-PD-(L)1 monotherapy and 4 with ICI-chemotherapy (carbo/pem/pembro). The incidence of any grade irAEs was 25% in both ICI monotherapy [grade 3 pneumonitis (*n* = 2)] and ICI-chemotherapy [grade 1 skin rash (n = 1)] populations (Table 5). The ORR for anti-PD-(L)1 monotherapy (*n* = 8) in this patient population in the second-line and beyond setting was 13% (1 CR). The ORR for chemotherapy and ICI therapy (*n* = 4) in the first-line setting was 75% (3 PR). The predominant tumor type in the HBV/HCV cohort was HCC including 17 patients treated with anti-PD-(L)1 monotherapy. The ORR in this subset of patients (*n* = 16) was 19% (3 PR) and any grade irAEs were noted in 44% patients (rash/pruritus (*n* = 6), hepatitis (*n* = 3) and diabetes mellitus (n = 1). The incidence of grade ≥ 3 was 25% (hepatitis (*n* = 2), rash (n = 1) and diabetes mellitus (n = 1). Although 2 patients developed grade 3 hepatitis, both had grade ≥ 2 hepatitis at baseline before ICI initiation.

**Table 5:** Subgroup analysis of efficacy and safety according to tumor type and ICI therapy

Tumor Types (N)	Co-infection (N)	Anti-PD-(L)1 Monotherapy			Anti-PD-(L)1 plus Chemotherapy		
		ORR^a^ N (%)	Any grade irAEs N (%)	Grade ≥ 3 irAEs N (%)	ORR N (%)	Any grade irAEs N (%)	Grade ≥ 3 irAEs N (%)
NSCLC (22)	HIV (12)	1(13)	2(25)	2(25)	3(75)	1(25)	0
	HBV/HCV(10)	1(14)	4(57)	2(29)	2(67)	1(33)	1(33)
HCC (17)	HBV/HCV(16)	3(19)	7(44) ^b^	4(25)^b^			

^a^Response evaluable patients

^b^Two patients with baseline grade ≥ 2 hepatitis

## Discussion

ICI therapy has reshaped the landscape of treatments in a broad array of cancers. Patients with chronic viral infection such as HIV, HBV, and HCV have been historically excluded from clinical trials of ICIs. Therefore, the efficacy and safety profile of ICI therapy has been largely unexplored, limiting physician’s ability to make informed treatment decisions for these patients. Here we report the results from our retrospective study of cancer patients with chronic viral infection treated with ICI therapy, which is one of the largest case series to date.

In the HIV cohort, in line with previous studies [12, 14], ICI therapy did not appear to adversely affect CD4+ T-cell counts or HIV viral load, although the number of patients with paired pre- and post-treatment values was small. Early evidence suggests that CD4+ T-cell counts may increase with PD-1 monotherapy [12, 14, 20]. Ongoing trials of ICI therapy in HIV-infected patients (NCT03304093, NCT03094286, NCT02595866, NCT02408861) are expected to shed light on the anti-viral efficacy of ICI therapy. The incidence of grade 3 or higher irAEs was 14%, which is comparable to the results from a recently published systematic review and a phase I trial of pembrolizumab [12, 14]. Chemotherapy plus ICI therapy – a treatment regimen that is being increasingly utilized in certain types of cancer such as NSCLC – did not seem to increase the risk of irAEs in patients with HIV infection, though this should be verified in future studies. ICI therapy showed anti-tumor activity with an ORR of 25%. In patients with NSCLC which is one of the most common non-AIDS defining cancers in HIV-infected patients [21]; 3 of 4 patients (75%) responded to anti-PD-1 and chemotherapy treatment in the first-line setting and 1 of 7 (13%) had a partial response to anti-PD-1 monotherapy in the second-line setting and beyond. Of note, responders included those with low a CD4 T-cell count. These efficacy results are largely consistent with those from landmark trials that excluded patients with HIV infection [2, 3, 22, 23].

In the HBV/HCV cohort, among the 6 patients with known pre- and post-treatment viral titers (2 HCV and 4 HBV), there was no evidence of viral reactivation. This is in line with the results from clinical trials of anti-PD-1 therapy in patients with HCC [17, 18]. Grade 3 or higher irAEs and ORR were similar to those observed in the clinical trials of anti-PD-1 therapy. As with the HIV cohort, combined chemotherapy and ICI therapy did not seem to increase the risk of irAEs, though definitive conclusion could not be drawn due to the small number of patients treated with the combination.

Several studies have shown that upregulation of PD-1 is associated with virus-specific CD8+ T-cell functional exhaustion in patients with HIV, HBV, or HCV infection [24–26] and PD-1/PD-L1 blockade restored the function of exhausted virus-specific CD8+ T cells in a preclinical model [27], providing a rationale for assessing antiviral effects of immunotherapy targeting the PD-1/PD-L1 pathway. However, it is unclear if anti-PD-(L)1 monotherapy alone would constitute a treatment strategy for chronic viral illness. For example, conflicting data exist on the anti-viral efficacy of anti-PD-1 therapy in patients with cancer [28–30]. Studies have shown that other immune checkpoints such as TIGIT, LAG-3, and TIM-3 may play a role in promoting tumor immune evasion and exhaustion of virus-specific T cells [31–34], suggesting that combination ICI therapy may need to be explored to effectively treat both cancer and chronic viral infection. Improving our understanding of pathways establishing viral latency and tumor resistance to ICI therapy will be critical to the rational development of immunotherapy in patients suffering from cancer and chronic viral illness.

Our study has several limitations. First, important viral parameters (e.g. CD4+ T-cell count, viral titer, antibody titer) were not collected in the majority of patients, restricting our ability to fully elucidate the anti-viral efficacy of ICI therapy in the patients included in the study. Monitoring of HIV, HBV and HCV viral load as well as CD4 count for PLWH during cancer treatment is necessary for patient safety and should be part of standard care for these patients. Second, while tumor response was able to be assessed in most patients, tumor assessment was not performed consistently, and some patients did not have imaging after initiation of ICI therapy, mainly due to clinical deterioration or loss to follow up. Despite these limitations, we believe that this case series provides evidence to help oncologists and their patients to inform decisions regarding the application of ICI therapy.

In summary, in this case series, we find that toxicity and efficacy rates were similar to those observed in patients without chronic viral infections, supporting the use of ICI therapy in this patient population and the inclusion of such patients’ in future ICI-based trials. Viral reactivation was not observed among HIV or HBV/HCV patients and anti-tumor activity was seen with anti-PD-(L)1 therapy alone or in combination with chemotherapy. Prospective studies are needed to validate these findings.

## Conclusions

In this case series of cancer patients with HIV, HBV, or HCV infection treated with ICI therapy including chemotherapy plus immunotherapy, we found that the safety and efficacy profile of ICI therapy is similar to that observed in those without chronic viral illness. These results suggest that ICI therapy is a safe and effective treatment option for patients with HIV, HBV, or HCV infection suffering from advanced-stage cancer.

## Supplementary information

**Additional file 1: Table S1.** Changes in HIV viral loads and CD4 T-cell counts during treatment with ICI

## Data Availability

The datasets used and/or analyzed during the current study are available from the corresponding author on reasonable request.
