# Supplementary material for: Safety and efficacy of immune checkpoint inhibitors (ICIs) in cancer patients with HIV, hepatitis B, or hepatitis C viral infection
Source: J Immunother Cancer. 2019 Dec 17;7:353. doi: 10.1186/s40425-019-0771-1 (PMC6918622; doi:10.1186/s40425-019-0771-1)
Supplement: Supplementary file 1 — Additional file 1: Table S1. Changes in HIV viral loads and CD4 T-cell counts during treatment with ICI [file 40425_2019_771_MOESM1_ESM.docx]

**Additional file 1: Table S1. Changes in HIV viral loads and CD4 T-cell counts during treatment with ICI**

|  | Type to ICI Therapy | HIV Viral Load before ICI therapy (copies/ml) | CD4+ T-cell Counts before ICI therapy (cells/ul) | HIV Viral Load during or after ICI Therapy (copies/ml) | CD4+ T-cell Counts during or after ICI Therapy (cells/ul) |
| --- | --- | --- | --- | --- | --- |
| Pt 1 | ICI plus chemotherapy | 111,000 | 530 | 7960 | 611 |
| Pt 2 | PD-1 monotherapy | 0 | 328 | 0 | 293 |
| Pt 3 | ICI plus chemotherapy | 0 | 616 | 0 | 366 |
| Pt 4 | PD-1 monotherapy | 0 | 96 | 81 | 61 |
| Pt 5 | ICI plus chemotherapy | 56572 | 77 | 82 | 93 |
